# An aPPARent Functional Consequence in Skeletal Muscle Physiology via Peroxisome Proliferator-Activated Receptors

**DOI:** 10.3390/ijms19051425

**Published:** 2018-05-10

**Authors:** Wendy Wen Ting Phua, Melissa Xin Yu Wong, Zehuan Liao, Nguan Soon Tan

**Affiliations:** 1 School of Biological Sciences, Nanyang Technological University 60 Nanyang Drive, Singapore 637551, Singapore; wphua003@e.ntu.edu.sg (W.W.T.P.); mwong018@e.ntu.edu.sg (M.X.Y.W.); liao0058@e.ntu.edu.sg (Z.L.); 2 Lee Kong Chian School of Medicine, Nanyang Technological University, 50 Nanyang Avenue, Singapore 639798, Singapore; 3 NTU Institute for Health Technologies, Interdisciplinary Graduate School, Nanyang Technological University, 50 Nanyang Drive, Singapore 637553, Singapore; 4 Institute of Molecular and Cell Biology, A*STAR, 61 Biopolis Drive, Proteos, Singapore 138673, Singapore; 5 KK Women’s and Children’s Hospital, 100 Bukit Timah Road, Singapore 229899, Singapore

**Keywords:** peroxisome proliferator-activated receptor, skeletal muscle, lipid metabolism, insulin resistance, aging, physical exercise, type 2 diabetes, muscle regeneration

## Abstract

Skeletal muscle comprises 30–40% of the total body mass and plays a central role in energy homeostasis in the body. The deregulation of energy homeostasis is a common underlying characteristic of metabolic syndrome. Over the past decades, peroxisome proliferator-activated receptors (PPARs) have been shown to play critical regulatory roles in skeletal muscle. The three family members of PPAR have overlapping roles that contribute to the myriad of processes in skeletal muscle. This review aims to provide an overview of the functions of different PPAR members in energy homeostasis as well as during skeletal muscle metabolic disorders, with a particular focus on human and relevant mouse model studies.

## 1. Skeletal Muscle

Skeletal muscle is the largest metabolic organ in the human body, and it contributes ~40% of the total human body mass in healthy non-obese adults. Beyond its well-recognized role in physical movement and postural stabilization, the importance of skeletal muscle in the whole-body metabolism has been increasingly acknowledged, as it can impact overall health and quality of life [1]. Skeletal muscle is a heterogeneous tissue composed of different fiber types, and it exhibits high metabolic flexibility when adapting to metabolic or energy demands, as well as prevailing conditions and activities. Skeletal muscle can withstand massive and sudden changes, both mechanically and bioenergetically, from rest to rapid contractile activity, because it has effective mechanisms for coping with ATP consumption and re-synthesis. While skeletal muscle is anatomically fixed at birth in mammals, postnatal muscle growth can undergo cellular changes, such as increases in length and girth, and some myofibers can experience changes in contractile activity and humoral factors in response to the nutrient availability [2]. The mammalian skeletal muscle can be classified across a spectrum, according to its contractile and metabolic properties, but it is broadly classified into two categories, namely, slow-twitch type I fibers and fast-twitch type II fibers. Slow-twitch type I fibers are rich in mitochondria and have a higher insulin sensitivity and glucose transporter 4 (GLUT4) expression levels than the fast-twitch type II fibers [3]. The type I fibers are rich in myoglobin, surrounded by many capillaries, and contain relatively abundant intracellular lipid levels for oxidative metabolism. These characteristics support long-duration contractile activities, such as walking and postural stabilization. In contrast, the fast-twitch type II fibers are large fibers with vast glycogen reserves that support their role in glycolytic metabolism. Type II fibers produce rapid contractions that are used for intense activities, but these fibers are easily fatigued. In mammals, type II muscle fibers can be further categorized into type IIa (fast-twitch oxidative), type IIb (fast-twitch glycolytic), and type IIx (an intermediate type between IIa and IIb). However, type IIb fibers are not detectable in the human skeletal muscle [4]. Muscle fiber type switching and tissue remodeling can occur on demand during exercise or during obesity and metabolic-related diseases. In response to exercise training, the metabolic phenotype of the muscle that is used changes along with the increase in size and strength. At rest, a trained muscle uses more energy from fat and less from carbohydrates than the untrained muscle [5]. Skeletal muscle is the predominant site of the insulin-mediated glucose uptake. The deregulation of skeletal muscle energy homeostasis plays a major role in the pathogenesis of peripheral insulin resistance and type 2 diabetes mellitus (T2DM). T2DM is characterized by chronic hyperglycemia, as a result of inefficient pancreatic beta-cell insulin secretion compensation. T2DM is also characterized by a chronic increase in plasma free fatty acid (FFA) levels and dyslipidemia. Excessive triglyceride accumulation in skeletal muscle, both the intramuscular and intramyocellular deposition, induces lipotoxicity, reduces glucose uptake, and ultimately leads to insulin resistance and T2DM [6]. Physiologically, the deregulation of the metabolic homeostasis in skeletal muscle causes muscle fiber type switching, from the slow-twitch to fast-twitch, as the disease worsens over time [7]. Understanding the changes in skeletal muscle during obesity and T2DM development is thus crucial for elucidating the underlying causes of insulin resistance.

The peroxisome proliferator-activated receptors (PPARs) have emerged as the master regulators of both lipid and glucose homeostasis, and are considered as valuable pharmaceutical targets for treating metabolic dysfunctions and T2DM. PPARs are ligand-activated transcription factors that belong to the nuclear hormone receptor superfamily, and they are activated by a variety of synthetic ligands and endogenous ligands, such as the naturally occurring FFAs and their metabolites, arachidonic acid and eicosanoids. The synthetic ligands of PPARs have been used successfully to treat T2DM and dyslipidemia. Specifically, thiazolidinediones (TZDs), such as rosiglitazone and pioglitazone, are specific PPARγ activators and are used as insulin sensitizers in order to improve insulin resistance in T2DM patients. Fibrates include fenofibrate, clofibrate, and ciprofibrate, which exhibit a predominant PPARα activity and induce lipid uptake and oxidation. The PPARα agonist clofibrate has been used to treat dyslipidemia. Insulin-sensitizing effects can also occur as a consequence of PPARα and PPARβ/δ activation. Physiologically, the members of the PPAR family also modulate basic processes, such as proliferation, differentiation, and postnatal development [8,9]. In this review, we will focus on the metabolic regulatory roles of PPARs in the skeletal muscle during healthy and diseased states, primarily with studies that have used human and mouse models.

## 2. Transcription Regulation by PPARs

Three related PPAR members, each encoded by distinct genes, have been identified and designated as PPARα, PPARβ/δ, and PPARγ. PPARγ has two distinct isoforms, PPARγ1 and PPARγ2. PPARγ2 is predominantly expressed in adipose tissue and is 30 amino acids longer than the PPARγ1 at the N-terminal [10]. As with most nuclear receptors, PPARs share modular structural characteristics. The N-terminal A/B domains encode the activation function 1 (AF-1), the C-domain consisting of the DNA binding domain (DBD), the D-domain, or the hinge domain that provides structural flexibility, and the E-domain containing the ligand binding domain (LBD) and the ligand-dependent activation function 2 (AF-2). Of the PPAR members, the LBDs of PPARα and PPARγ are the most similar in shape and size, whereas the LBD of PPARβ/δ is significantly smaller [11,12]. The differences in amino acid sequences among the PPAR members also indicate that the LBD pocket of PPARα is more lipophilic than that of the two others. These structural differences among the PPAR LBDs suggest the influences of the structurally distinct ligands with varying binding affinities that contribute to ligand selectivity [13]. All members of PPAR form obligate heterodimers with retinoid X receptors (RXRs) and bind as a complex to the consensus sequences, known as peroxisome proliferator response elements (PPREs), located in the regulatory region of their target genes.

In addition to ligand binding, the activity of PPARs is also affected by post-translational modifications, such as phosphorylation, SUMOylation, and ubiquitination, as well as through regulatory proteins, such as AMP-activated protein kinase (AMPK) and cryptochrome (CRY1). Regulation by insulin and insulin-induced PPAR phosphorylation has been reported to enhance the PPAR transcriptional activity [14]. Post-translational modification by ubiquitination has been shown to be affected by the presence of the PPAR ligand. In the absence of the ligand, PPARα and PPARβ/δ are poly-ubiquitinated and targeted for subsequent degradation [15,16]. The presence of PPARγ agonists, on the other hand, enhances the PPARγ polyubiquitination, which promotes its degradation. The monoSUMOlyation of PPARα and PPARγ has been reported, in which the transcriptional activities of both PPARs are inhibited [17]. The role of energy metabolism and circadian regulation in skeletal muscle has recently been understood through the modulation of the PAR protein. Recent studies by Jordan et al. (2017), on skeletal muscle circadian rhythm, have shown that the circadian transcriptional repressors CRY1 and CRY2 function as co-repressors of PPARβ/δ, possibly via an AMPK-dependent signaling pathway [18]. Collectively, the post-translational regulation of the PPAR protein has a direct impact on the cellular metabolism and energy production.

PPARs are diverse regulators that fundamentally regulate the energy metabolism at the transcription level. Each member displays distinct tissue distribution patterns and pharmacological profiles. PPARα is highly expressed in active metabolic tissues, such as the liver, kidney, heart, and skeletal muscle [19], whereas PPARγ is expressed in primarily the white and brown adipose tissue, where most of the free fatty acids are deposited [20]. PPARβ/δ is ubiquitously expressed because of its importance in the systemic and basic cellular functions, which include the energy modulation in metabolically active tissues, inflammation, wound healing, and keratinocyte and intestinal cell differentiation [7,9]. The *PPARD* gene ablation in mice results in a high embryonic lethality [21], and the PPARγ-deficient mice exhibit an embryonic lethality by E10 [22]. These findings highlight the importance and complex physiological roles of PPARs. All three of the PPARs are expressed in the skeletal muscle at different amounts, as follows: PPARβ/δ has the highest expression levels, followed by PPARα and PPARγ [23,24,25].

## 3. Nutrient Sensing by PPARs

Members of the PPAR family modulate metabolic responses through sensing and responding to fluctuations in the nutrient availability. Major dietary constituents, such as fatty acids and carbohydrates, can regulate the gene expression of several metabolic pathways via hormones and PPARs and, in turn, induce their utilization [26]. In a post-prandial state, the availability of metabolic precursors promotes the synthesis of natural PPAR ligands and induces PPAR trans-regulation so as to promote anabolism and storage. Upon nutrient scarcity, PPARs are directly activated by the release of FFAs from lipid reserves, and they stimulate the transcription of genes that are involved in FFA uptake and fatty acid oxidation in the skeletal muscle, as well as glycogenolysis, gluconeogenesis, and ketone body synthesis in the liver, reviewed in [27].

Nutrient intake and energy metabolism are closely associated and are subject to hormonal regulation. Insulin, one of the main hormones that regulates whole-body metabolism, promotes glucose uptake in the metabolically active tissues, such as the liver, fat, and skeletal muscle. During post-prandial state, insulin is secreted from the pancreatic beta cells into the bloodstream in response to increased blood glucose levels. At the peripheral tissues, such as skeletal muscle, insulin binds to the insulin receptors at the plasma membrane in order to trigger the insulin signaling cascade via insulin receptor substrate 1 (IRS1) phosphorylation, protein kinase B (AKT/PKB) activation, and glucose transporter type 4 (GLUT4) translocation to the plasma membrane [28]. These actions promote an extracellular glucose clearance [29]. Skeletal muscle accounts for over 80% of the insulin-dependent glucose uptake [30]. Glucose serves as an immediate source of energy and is subsequently converted into acetyl-coenzyme A (acetyl-CoA), by the pyruvate dehydrogenase complex (PDC). Then, it is channeled into the tricarboxylic acid (TCA) cycle and undergoes oxidative phosphorylation in the mitochondria [31]. In skeletal muscle, the excess glucose is stored as glycogen or used as a precursor for lipid synthesis [31]. As blood glucose levels drop over time, the body transits from a fed to fasted state, triggering the change from glucose to free fatty acids (FFAs) as the preferred fuel substrates of the skeletal muscle and liver. This dynamic glucose-FFA cycle, also known as the Randle cycle, provides metabolic flexibility and survival adaptation so as to conserve the whole-body glucose supply and is of major quantitative importance in the skeletal muscle, as reviewed in [32,33].

During fasting, both PPARα and PPARβ/δ are upregulated in the skeletal muscle in rodents [34], but only PPARβ/δ is upregulated in the human skeletal muscle [7,35]. Upon the increased FFA influx, the FFAs are hydrolyzed into acyl-CoA complexes, which are then channeled into the mitochondria by the carnitine palmitoyltransferase I (CPT1) for fatty acid oxidation. One of the key genes that regulates the glucose-FFA shuttle is the pyruvate dehydrogenase kinase (PDK), which is a classical PPAR target gene. PDK inactivates PDC, via phosphorylation, and reduces oxidation of the glycolysis-derived pyruvate. These effects decrease the glucose utilization in order to conserve glucose. In human skeletal muscle, all four of the PDK isozymes are PPARβ/δ target genes, and PDK2 and PDK4 are the most abundantly expressed [35,36]. In the skeletal muscle of PPARβ/δ knockout mice, PDK4 expression is markedly blunted [37]. Interestingly, the PDK4 expression is unaffected in the skeletal muscle of the fasted PPARα knockout mice [25]. These findings thus suggest that PPARβ/δ is the primary PPAR member that regulates the skeletal muscle substrate utilization.

## 4. Regulation of Lipid Metabolism in Skeletal Muscle by PPARs

Fat and excess calories from the diet are converted into the concentrated form of triglycerides to store metabolic energy over extended periods of time. Triglycerides are typically stored in three main organs (ranked in order, from the greatest to least amount stored), namely, adipose tissue, skeletal muscle, and liver [38]. During fasting or increased energy demands, triglycerides in adipose tissue are hydrolyzed into FFAs and delivered to tissues through the action of lipoprotein lipase (LPL), and can be used either for fatty acid β-oxidation in the energy-converting mitochondria or as building blocks for cellular functions and signaling.

Over the past decade of research, PPARs have emerged as master regulators of the lipid metabolism. In humans, skeletal muscle accounts for more than 30% of the total energy expenditure, and up to 70% of this energy is derived from FFAs in resting muscle. Of the three PPAR members, PPARα and PPARβ/δ play central roles in regulating lipid homeostasis [25]. PPARγ promotes glucose uptake in skeletal muscle, in order to play a role in insulin-stimulated glucose metabolism [39]. In vivo, PPARα and PPARβ/δ regulate the genes that are involved in FFA uptake, such as cluster of differentiation 36/SR-B2 (CD36) and LPL; FFA intracellular transport, such as fatty acid binding protein 3 (FABP3); and fatty acid oxidation, such as CPT1 and stearoyl-CoA desaturase (SCD). The genes that are involved in fatty acid oxidation and utilization are similarly regulated by PPARα and PPARβ/δ in skeletal muscle, as shown by overexpression studies [40,41,42]. Skeletal muscle-specific PPARβ/δ overexpression also induces characteristic shifts towards oxidative fibers and increased oxidative capacity [7]. Conversely, selective PPARβ/δ ablation in skeletal muscle leads to lower oxidative capacity in the fibers, resulting in obesity and T2DM [43]. In contrast to PPARβ/δ, PPARα overexpression promotes fiber type shifts towards glycolytic type II fibers, and these fibers are protected from diet-induced obesity. Interestingly, when fed a high-fat diet, PPARα-overexpressing mice have significantly higher intramuscular triglyceride concentrations than control mice, and they develop glucose intolerance [41]. In PPARα knockout mice, however, fatty acid oxidation is reduced during starvation despite an increase in oxidative fibers [25].

### 4.1. Regulation of Lipid Transport in Skeletal Muscle by PPARs

**Figure 1:**
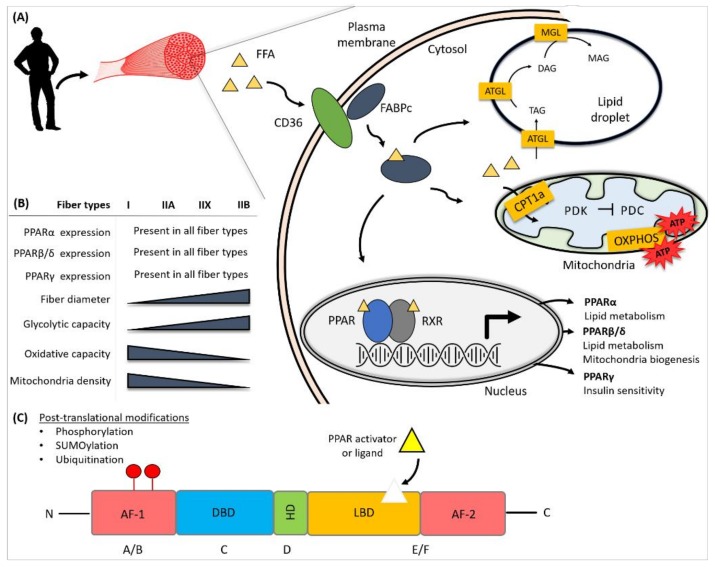
Schematic diagram of skeletal muscle fiber and its fatty acid handling. (**A**) The fate of free fatty acid (FFA) in skeletal muscle. FFA uptake is mediated by receptors, such as CD36, at the plasma membrane. Within the cell, FFA is transported throughout the cellular compartments, via the lipid transporter, FABPc. FFAs can either be targeted to the lipid droplet for storage, translocated to the mitochondria for fatty acid oxidation, or serve as a ligand for peroxisome proliferator-activated receptors (PPARs) within the nucleus. In the skeletal muscle, PPARα and PPARβ/δ are mainly involved in lipid metabolism regulation. PPARβ/δ is also involved in regulating mitochondria biogenesis while PPARγ is involved in skeletal muscle insulin sensitivity and glucose regulation. (**B**) The spectrum of skeletal muscle fiber type characteristics. All three of the PPAR isotypes are expressed regardless of the fiber types. Slow-twitch type I fibers are smaller in fiber diameter, with high oxidative capacity and mitochondria density, while fast-twitch type II fibers have a range in their fiber diameters, typically higher glycolytic capacity with lower mitochondria density, and oxidative capacity in comparison to type I fibers. (**C**) Schematic diagram of PPAR protein structure. PPARs are regulated by post-translational modifications, such as phosphorylation, SUMOylation, and ubiquitination in the presence or absence of ligand. Activation function, AF; DNA-binding domain, DBD; Hinge domain, HD; ligand binding domain, LBD.

Unlike glucose, which is water soluble, circulating FFAs are usually associated with albumin or exist as fatty esters and phospholipids in lipoproteins. On the plasma membrane surface, LPL mediates the hydrolysis of triglyceride-rich lipoproteins. This hydrolysis releases the encapsulated lipids and is thus considered a rate-limiting step for lipid uptake. The cellular lipid uptake was initially thought to occur via passive diffusion because of the hydrophobic nature of the plasma membrane. However, it is now widely recognized that FFA uptake requires a highly regulated, protein-mediated action by the transporter proteins. In humans and rodents, CD36, FABPs, and fatty acid transport proteins (FATPs) are co-expressed in the skeletal muscle, which is key in facilitating FA transport, and their expression levels are regulated predominantly by PPARβ/δ [7] (Figure 1). Approximately 70% of total FFA uptake is mediated by CD36 [44], although the mechanisms of FFA transmembrane movement and the binding specificity of CD36 are not understood [45]. It has been suggested that CD36 promotes fatty acid partitioning at the outer leaflet for translocation through the lipid bilayer and that it provides a docking site for FABPs and other enzymes at the intracellular side of the membrane, so as to facilitate the transport of the incoming FFAs [45]. Cytoplasmic FABP (FABPc) serves as an acceptor for FFAs, shuttles them through the cellular compartments, and protects against lipotoxic accumulation and aggregation within the cell [46]. The fatty acid transporters in skeletal muscle exhibit different capacities for FFA transport and metabolism. An in vivo study of CD36, plasma membrane FABP (FABPpm), FATP1 or FATP4 overexpression in the anterior tibialis muscle of rats showed the differential effects on FFA transport and utilization in skeletal muscle [47]. The authors have reported that CD36 and FATP4 are quantitatively the most effective in FFA transport. Interestingly, the transporter overexpression did not alter the rates of FFA esterification into triglycerides, but it increased fatty acid oxidation that was observed with CD36 and FABPpm overexpression [47,48] (Figure 1).

Mammalian FABPs are small skeletal muscle proteins of approximately 15 kDa and are involved in the reversible binding of FFAs, in order to facilitate trafficking to various cellular compartments, such as peroxisomes, mitochondria, and nuclei. In humans, there are nine FABP isoforms (FABP1-9) that are differentially expressed in metabolically active tissues [49]. In adult skeletal muscle, FABP3 is predominantly expressed and is more abundant in type 1 oxidative fibers than in type 2 glycolytic fibers. FABP3 is responsible for FFA shuttling to the outer mitochondrial membrane, where FFAs are converted into their acyl-CoA derivatives by acyl-CoA synthetase, and are subsequently channeled for mitochondrial β-oxidation [50,51]. A small amount of acyl-CoA is converted into lipid intermediates, such as ceramide, diacylglycerol, and phospholipids, which can function as lipid secondary messengers or modulate membrane structures [52,53]. FABPs have been shown to interact with PPARs in the nucleus, so as to mediate transcriptional activities. Recently, the ligand-sensitive nuclear localization signal in FABP5 protein conformation has been described. In this conformation, FABP5 facilitates PPARβ/δ transcriptional activation through nuclear trafficking of linoleic acid and arachidonic acid [54]. Similar reports have shown that FABP1, FABP2, and FABP3 can increase FFA shuttling to the nucleus and enhance ligand-mediated PPARα transactivation [51,55,56], whereas PPARγ agonists can induce the nuclear localization of FABP4 [57,58]. However, the specificity of the lipid species with FABP chaperone activity and the significant impact of each FABP isoform on the transcriptional regulation in the skeletal muscle remains unclear.

### 4.2. Regulation of Muscle Lipolysis by PPARs

Lipolysis is the process through which FFAs are sequentially hydrolyzed. Lipolysis is first mediated by the rate-limiting enzyme adipose triglyceride lipase (ATGL), which hydrolyzes triglyceride to diacylglycerol and one fatty acid molecule. Diacylglycerol is then converted into monoacylglycerol, by hormone-sensitive lipase (HSL). The final step of FFA hydrolysis by monoacylglycerol lipase (MGL) produces glycerol and the third fatty acid molecule. In the mouse skeletal muscle, lipolysis can occur without stimulation (basal conditions) or with hormone stimulation [59]. Under either condition, ATGL and HSL collectively account for most of the hydrolysis activity [60]. ATGL is an evolutionarily conserved enzyme for fat storage lipolysis [61]. It is highly expressed in type I fibers in both mice and humans and is a reported transcriptional target of PPARα in rodents [62]. ATGL knockout mice have a shorter life-span and exhibit defective lipolysis and increased triglyceride accumulation in non-adipose tissues, including skeletal muscle [63,64]. These mice also show a concomitant decrease in muscle relaxation and have an increased reliance on carbohydrates as the major fuel source at rest [65]. Interestingly, pharmacological treatment of ATGL knockout mice with PPARα agonists reversed the excessive systemic lipid accumulation, improved metabolic flexibility in substrate switching from glucose to fatty acids, and prevented premature death [64]. ATGL overexpression in different muscles has varied effects on skeletal muscle fatty acid oxidation in mice. These varied effects are most likely due to the differential expression levels of ATGL among fiber types. Increased fatty acid oxidation was reported upon ATGL overexpression in the soleus muscle via electroporation [62]. However, adenovirus-mediated ATGL overexpression in the tibialis anterior muscle was not sufficient to alter fatty acid oxidation rates [66]. Similarly, mutations in the *PNPLA2* gene, which encodes ATGL in humans, can lead to neutral lipid storage diseases with myopathy. In humans, ATGL is exclusively expressed in type I muscle fibers and plays an important role in skeletal muscle FA turnover [67]. ATGL deficiency in young adults resulted in increased lipid accumulation in primarily type I skeletal muscle [68]. However, treatment with a PPARα agonist was less successful in humans than in rodents [69].

### 4.3. Regulation of Muscle Lipid Storage by PPARs

Skeletal muscles stockpile excess FFAs in lipid droplets as an energy reservoir. These FFA stores are commonly referred to as intramuscular triglycerides. Excess FFAs are converted in the endoplasmic reticulum (ER) and stored in lipid droplets (LDs), which are surrounded by a phospholipid monolayer and LD-associated surface proteins. These LDs are dynamic structures that function as more than temporary fuel storage. In fact, they serve as a reserve pool of intracellular signaling mediators for ligands, such as PPAR, and are thought to have a protective mechanism against possible lipid aggregation that leads to lipotoxicity and ER stress after the excess uptake of FFAs and sterols. In skeletal muscle, lipid droplets are distributed between myofibrils (intermyofibrillar LDs) and beneath the plasma membrane (subsarcolemmal LDs). These LDs serve as transport organelles between cellular compartments and as a readily available energy pool for short-term or long-term muscular contractions. PPAR agonists have been reported to regulate LD-associated proteins, such as perilipins (PLIN1-5), in various organs. Perilipins, except PLIN1, are expressed in skeletal muscle in humans and rodents [70]. PLIN2, one of the most abundantly expressed LD-coating proteins in skeletal muscle, is thought to maintain insulin sensitivity in skeletal muscle and promote the storage of FFAs in the form of triglycerides [71]. PLIN2 is induced upon PPARβ/δ activation by GW501516 in both human primary myocytes and mouse skeletal muscle [72,73]. In PPARα knockout mice, PLIN2 and PLIN5 expression levels are decreased in the soleus, whereas PLIN3 and PLIN4 expression levels seem to be unaffected [74]. Interestingly, immunofluorescent staining of human and rodent skeletal muscle sections have shown that PLIN2 is abundantly expressed in type I fibers, which contain more intramuscular triglyceride contents than type II fibers [75,76]. Similarly, the direct regulation of PLIN5 by PPARβ/δ in the soleus and gastrocnemius of wild-type mice has been observed. In this study, a conserved PPRE in humans and mice had been found in the first intron of PLIN5 [74]. However, PLIN5 protein levels in the skeletal muscle did not seem to be altered in PPARβ/δ knockout mice.

PLIN5 has been suggested to regulate FFAs storage and to be involved in skeletal muscle adaptation in type II fibers, in response to exercise and fasting [77]. Similarly to PLIN2, the PLIN5 expression levels are higher in the oxidative fibers than in glycolytic fibers [78], and its protein levels are associated with intramuscular triglyceride levels in both rodents and humans [75]. In glucose-intolerant human subjects, it has been reported that PPARγ agonists can induce PLIN5 mRNA expression, and PLIN5 mRNA expression is negatively correlated with the body mass index (BMI) in non-diabetic subjects [79]. The role of PPAR regulation and its effects on perilipin functions in skeletal muscle physiology, however, need further investigation, as most of the studies on PLIN5 have been performed in vitro [46].

## 5. Regulation of Mitochondrial Biogenesis and Function by PPARs

The members of the PPARγ-coactivator 1 (PGC-1) family, such as PGC-1α and PGC-1β, regulate mitochondrial oxidative metabolism and biogenesis, and activate gene transcription through coordination with PPARα, PPARβ/δ, and other nuclear receptors. PGC-1α is reported to be a direct target of PPARβ/δ, but not PPARα, in the skeletal muscle, via agonism [43,80] and during conditions of increased energy demands, such as cold, exercise, and fasting [43,81]. Moreover, in vivo PPARβ/δ overexpression, via electroporation in adult rat muscle, caused an increase in PGC-1α protein levels [82]. PGC-1α thus mediates a positive feed-forward transcriptional control of the PPAR-regulated genes that are involved in fatty acid oxidation and carbohydrate metabolism, as well as an auto-regulatory loop, in which PGC-1α regulates its own gene expression [83]. Gene manipulation of PGC-1α and PGC-1β in skeletal muscle produces phenotypes similar to those of PPARβ/δ transgenic mice. Conversely, PGC-1α or PGC-1β overexpression in mouse skeletal muscle induces oxidative fiber development, promotes fatty acid oxidation and increases the capacity to sustain physical activity in mice [84,85]. However, PPARβ/δ overexpression in mice does not increase PGC-1α mRNA levels and does not affect mitochondrial function [42,86]. Additionally, transcription factors such as mitochondrial transcription factor A (TFAM) and mitochondrial transcription factors B1 (TFB1M) and B2 (TFB2M), which directly regulate mitochondrial biogenesis via nuclear respiratory factors (NRF1 and NRF2) are not known to be classic PPARβ/δ target genes [87]. Thus, the precise regulation of PPARβ/δ and PGC-1α in mitochondrial function and biogenesis has been a long-standing question. Recently, Koh et al. [88] used an electroporation-mediated PPARβ/δ overexpression in mouse muscles to demonstrate that PPARβ/δ modulates mitochondrial biogenesis and PGC-1α expression, in both a transcriptional manner and a posttranslational manner. PPARβ/δ overexpression in adult mice increases NRF1 and mitochondrial electron transport chain enzyme protein levels, before increasing PGC-1α protein levels. Moreover, PPARβ/δ decreased PGC-1α degradation via ubiquitin-proteasome system, through binding and blocking its ubiquitin-binding site. These actions led to the gradual accumulation of the PGC-1α protein [88]. The authors also reported the auto-regulation of PPARβ/δ, suggesting a feed-forward mechanism that is important in the mitochondrial oxidative metabolism and biogenesis.

## 6. Dysregulation of Lipid Metabolism and PPAR during Insulin Resistance and T2DM

Insulin resistance is the key pathophysiological feature of obesity and T2DM, and is caused by imbalances in insulin action in peripheral tissues, insulin secretion, or both. In skeletal muscle, the major causes of insulin resistance are thought to be the excess accumulation of intramyocellular lipid (IMCL) and the inhibition of one or several steps in the insulin signaling cascade [89]. IMCL includes all types of lipids within the myocytes. Myocytes are composed of mostly triglycerides, but also include the lipid intermediates of lipid metabolism, ceramides, diacylglycerol, phospholipids, and sphingolipids [90]. The most common cause of lipid accumulation is overnutrition, which leads to an increase in FFA uptake that exceeds the rates of fatty acid oxidation and storage [91]. High IMCL concentrations have also been negatively associated with insulin sensitivity in non-obese adults [92], high-fat diet rodent models [93], and lean offspring of T2DM patients [94]. Similarly, acute lipid overload in skeletal muscle decreases peripheral insulin sensitivity in healthy individuals [5,95]. Paradoxically, it has been reported that endurance athletes are highly insulin-sensitive, despite possessing higher IMCL concentrations than normal healthy individuals. This phenomenon is thus called the ‘athlete’s paradox’ [96]. These trained athletes, however, have a high capacity for fat oxidation and have high glucose disposal rates, but are not totally immune to lipid-induced insulin resistance [5,96]. Unlike obese individuals and T2DM patients, the turnover rates of IMCL in trained athletes is high, and this turnover is an adaptive physiological response rather than a pathological condition [5]. Thus, endurance athletes do not bear the ascribed toxic effects on insulin signaling.

Ceramide and diacylglycerol accumulation interfere with the insulin signaling cascade through the direct interaction with and activation of protein kinase C (PKC) isoforms, so as to reduce glucose uptake [91,97,98]. In the skeletal muscle, a 50% increase in endogenous ceramide levels, induced by treatment with a high concentration of saturated FFAs, is sufficient to inhibit AKT/PKB activity [99]. In obese insulin-resistant human subjects, ceramide concentrations were found to be nearly two-fold higher in muscle compared with lean insulin-sensitive human subjects [100]. In contrast, overexpressing acid ceramidase, which converts ceramide into sphingosine, fully negates the inhibitory effects of high FFA treatment on insulin signaling [101]. Additionally, ceramide has also been shown to stimulate protein phosphatase 2A (PP2A), a phosphatase long known to negatively regulate AKT/PKB [102]. The inverse relationship between ceramide and insulin sensitivity has been reviewed [97]. Furthermore, PKCθ activation by diacylglycerol, induces insulin resistance through inhibiting IRS1-associated phosphatidylinositol-3 kinase (PI3K) activity [103,104]. Diacylglycerol acyltransferase 1 (DGAT1), a downstream PPARβ/δ target gene, catalyzes the conversion of diacylglycerol and fatty acyl-CoA to triglyceride [105]. The skeletal muscle-specific DGAT1-overexpressing mice have low diacylglycerol concentrations and are protected from diet-induced insulin resistance, despite the increased FFAs accumulation in their skeletal muscle [106].

PPAR agonists have been of clinical interest since the discovery of fibrates and the TZDs for treating metabolic-related diseases. Below, we describe the impact of PPAR regulation in skeletal muscle, during insulin resistance and T2DM.

### 6.1. PPARγ Agonists and Insulin Resistance and T2DM Treatment

PPARγ ligands, including TZDs, have hypoglycemic effects, reduce insulin resistance, and improve insulin sensitivity. In the early 1980s, TZDs were reported as insulin sensitizers. Currently, pioglitazone is the only FDA-approved TZD for treating T2DM. This drug has lipid-modifying benefits and can reduce adverse cardiovascular outcomes. The insulin-sensitizing effects of TZDs can be attributed to the activation of skeletal muscle PPARγ. This activation maintains insulin signaling activity, even though PPARγ is expressed at low levels. Given the whole-body skeletal muscle mass, the regulation of the skeletal muscle PPARγ remains physiologically relevant. The direct action of TZDs on non-adipose tissues has been indicated in adipose tissue-specific PPARγ-silenced mice, in which TZD treatment improved insulin sensitivity in the skeletal muscle and the liver, despite an increase in triglyceride deposition [107]. In obese Zucker rats, short-term treatment with rosiglitazone increases the skeletal muscle tyrosine phosphorylation of insulin receptor and IRS-1, and induces AKT/PKB activation [108]. Similarly, muscle biopsies that were obtained from T2DM patients that were treated with either rosiglitazone or pioglitazone showed increased insulin-stimulated IRS-1 tyrosine phosphorylation, IRS-1-associated PI3-kinase activity, and AKT/PKB activity [109,110]. The TZD administration, however, has been reported to stimulate skeletal muscle glucose uptake acutely and improve glucose handling through a PPARγ-independent mechanism [111,112]. Moreover, the PPARγ-sparing TZD analogs have similar insulin-sensitizing pharmacological effects to rosiglitazone and pioglitazone in rodent models [113]. These results suggest that the insulin-sensitizing effects of TZDs may be independent of PPARγ regulation, to some degree. Despite the varied pharmacological actions of TZDs via PPARγ regulation, the role of PPARγ in the skeletal muscle in glucose homeostasis and insulin sensitivity remains physiologically and clinically relevant. In the human skeletal muscle, PPARγ expression is acutely regulated and increased by insulin [114]. PPARγ activation directly regulates the expression of the glucose transporters GLUT1 and GLUT4, and promotes their translocation to the cell surface so as to increase the cellular glucose uptake. In addition, GLUT4 regulation by PPARγ is remarkably conserved across the vertebrate evolution, from fish to mammals [115]. In L6 muscle cells, PPARγ agonists, but not PPARα agonist WY14643, have been shown to increase IRS1 protein expression directly [116]. Moreover, constitutive PPARγ activation in the mouse skeletal muscle decreases intramuscular lipid accumulation, induces a shift towards the oxidative fiber type, and protects against susceptibility to diet-induced insulin resistance [117]. Conversely, skeletal muscle-specific PPARγ knockout mice have an increased adiposity and are glucose intolerant and insulin resistant [118,119]. However, the young skeletal muscle of PPARγ-deficient mice remained responsive to the TZD treatment, despite a high-fat diet-induced hepatic insulin resistance and excess adiposity [119]. These findings led to the suggestion of age-dependent differences in TZD insulin-sensitizing effects and the potential role of tissue crosstalk in the regulation of whole-body insulin sensitivity [120]. In humans, dominant negative PPARγ mutations are associated with obesity [121], dyslipidemia, and severe insulin resistance [122], whereas a common polymorphism (Pro12Ala) has been shown to decrease PPARγ receptor activity, improve insulin sensitivity, and decrease T2DM risk [123,124].

### 6.2. PPARα Agonists and Insulin Resistance and T2DM Treatment

PPARα plays a pivotal role in the liver during the nutritional transitions and intricately controls hepatic lipid metabolism and whole-body glucose homeostasis [27]. The role of skeletal muscle PPARα in regulating the insulin signaling pathway is, however, less clear. Though PPARα has metabolic regulatory roles, its expression in skeletal muscle remains unchanged during fasting [35]. The clinical use of fibrates for treating hyperlipidemia in obese individuals and T2DM patients was first approved in the late 1960s [125]. The fibrates that are commonly used for clinical treatment are bezafibrate, fenofibrate, and gemfibrozil. Fenofibrate treatment in patients with metabolic syndrome improves lipid profiles and increases insulin sensitivity [126,127]. Recently, bezafibrate has been reported to increase skeletal muscle AKT/PKB phosphorylation and improve the insulin sensitivity in insulin-deficient streptozotocin-treated mice [128]. However, bezafibrate and fenofibrate exhibit weak PPARβ/δ and/or PPARγ agonist activity [125,129]. Therefore, the direct pharmacological activity of PPARα on human skeletal muscle insulin sensitivity requires further investigation.

### 6.3. Evidence for PPARβ/δ Agonist Treatment of Insulin Resistance and T2DM

PPARβ/δ agonists may be insulin sensitizers and have been suggested as a therapeutic approach for treating metabolic dysfunction and T2DM. Currently, there are no PPARβ/δ agonists that are approved for clinical treatment, but several are in the development and clinical study phases [8]. One prominent PPARβ/δ-selective agonist is seladelpar (MBX-8025), which is currently in clinical phase 2/3 for primary biliary cirrhosis, and has previously been shown to improve the insulin sensitivity and dyslipidemia in overweight subjects [130]. The well-known GW501516, though its development was halted in 2007, has since served as an important PPARβ/δ-specific agonist in the elucidation for PPARβ/δ physiological and pathophysiological functions. In animal models of obesity and T2DM, PPARβ/δ activation, through specific agonists or genetic manipulation, ameliorates hyperglycemia, insulin resistance, and dyslipidemia. PPARβ/δ silencing renders mice glucose intolerant and less metabolically active [131]. Similarly, the skeletal muscle-specific PPARβ/δ knockout mice exhibit insulin insensitivity and impaired glucose tolerance [43]. PPARβ/δ agonist treatment improves whole-body insulin sensitivity through complementary actions in the liver and skeletal muscle. In insulin-resistant ob/ob mice, activating PPARβ/δ through GW501516 ameliorates hyperglycemia-mediated glycolysis, and lipogenesis increases in the liver so as to reduce hepatic glucose output. Simultaneously, GW501516 promotes FAO in the skeletal muscle to enhance insulin sensitivity [131]. In addition, long-term GW501516 treatment in wild-type mice reduces body weight and circulating triglyceride levels [42].

## 7. Regulation of PPARs during Physical Exercise

**Figure 2:**
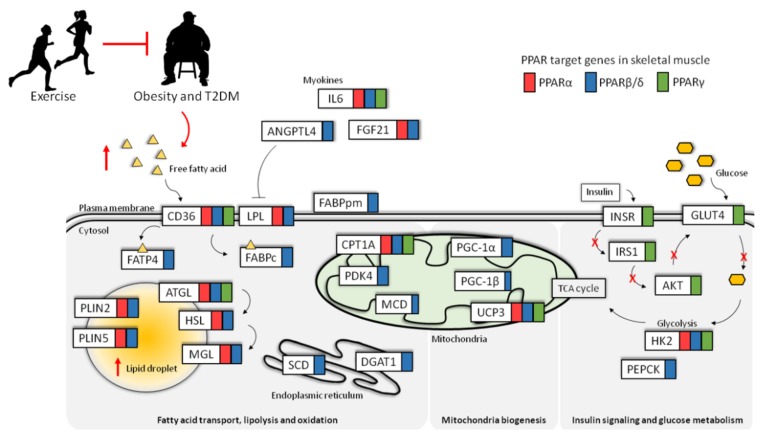
List of genes regulated by PPARα (red box), PPARβ/δ (blue box), and PPARγ (green box) in skeletal muscle. During obesity (red arrows), increased free fatty acid flux leads to excess lipid droplet accumulation, lipid dysregulation, and deregulation of insulin signaling and glucose uptake. Physical exercise can prevent obesity-related disorders and T2DM. Fibroblast growth factor 21, FGF21; malonyl-CoA decarboxylase, MCD; uncoupling protein 1, UCP1; insulin receptor, INSR; hexokinase 2, HK2; and phosphoenolpyruvate, PEPCK.

Adopting and maintaining physical activity is by far the best intervention and prevention for obesity and T2DM. Short-term aerobic exercise can increase glucose uptake by muscles during exercise and can increase insulin-mediated glucose storage in muscles after exercise [132]. In addition, both short-term exercise and endurance training have been reported to increase PPARβ/δ expression levels in both human and rodent muscles [73,133]. In obese and overweight humans, PPARβ/δ expression levels increase with exercise and are associated with the transcription of oxidative and lipoprotein metabolism genes, as well as PGC-1α [133] (Figure 2). In mice, endogenous PPARβ/δ activation with GW501516 treatment can enhance physical performance and upregulate oxidative genes, mitochondrial biogenesis, and fiber type switching [42]. A recent study showed that GW501516 promotes running endurance by preserving glucose. Activation of muscle PPARβ/δ coordinately reduces glucose catabolism to prevent hypoglycemia and facilitate a progressively longer running time [105]. Similarly, the authors also showed that overexpressing constitutively active PPARβ/δ in rodent skeletal muscle increased the running endurance of these transgenic mice [42]. Furthermore, in the mouse model of ischemic cardiomyopathy, the impaired exercise endurance following myocardial infarction could be reversed by the PPARβ/δ agonist GW501516 [134]. The pharmaceutical activation of PPARβ/δ has attracted much interest as an exercise mimetic to promote oxidative myofibers and running endurance without exercise. Despite a lack of evidence for its clinical safety, GW501516 has become an interest in endurance athletes because of its ability to influence energy expenditure and improve adaptations to training. Unfortunately, this drug has added complexity to the doping dilemma in competitive sports, which has culminated in the suspension of many athletes from the Olympics. The clinical development of PPARβ/δ agonists has been unsuccessful to date, and GW501516 remains a banned metabolic modulator by the World Anti-Doping Agency. Pharmaco-equivalents with better safety profiles, however, are still heavily researched [135,136].

Similarly to PPARβ/δ in humans, the expression levels of PPARα and its downstream target genes increase upon endurance training [133,137]. In skeletal muscle biopsies from spinal cord-injured subjects, the fiber type switching from type 1 oxidative fibers to type II glycolytic fibers often occurs as a result of muscle disuse, and PPARα expression is reduced [138]. In rodents, PPARα knockout mice are less tolerant of endurance exercise, although their skeletal muscle glycogen depletion rate is similar to their wild-type counterparts [25]. Interestingly, genetic variations in PPARα and PPARγ appear to play a role in athleticism. A recent study has found that *PPARA* gene intron 7 G/C polymorphism correlates to an endurance ability. Athletes with high levels of performance in endurance sports have a higher frequency of the GG genotype and G allele [139]. This genotype has also been associated with an increased skeletal muscle fatty acid β-oxidation rate and an increased proportion of type I slow-twitch fibers [140]. The *PPARG* Pro12Ala polymorphism, which is associated with an improved glucose utilization in skeletal muscles, is prevalent in Polish athletes who are involved in sports that involve short-term and intense exercises, such as power-lifters, weight-lifters, and throwers [141].

## 8. Regulation of Skeletal Muscle Regeneration by PPARs

Skeletal muscle injuries are among the most common soft tissue injuries [142,143], which occur not only during sports traumas and daily activities, but they are also a major concern of diabetic complications, such as muscle ischemia and peripheral vascular disease—the major risk factor of limb amputation in diabetic patients [144,145].

Skeletal muscle regeneration is initiated shortly upon injury and undergoes three main coordinated phases of healing—destruct, repair, and remodel [142]. Upon injury, ruptured myofibres first undergo necrosis, which induces an inflammatory reaction. The damaged tissues are then cleared by infiltrated immune cells, such as macrophages and neutrophils, through phagocytosis [146]. The activation and infiltration of the immune cells further promote the activation of myogenic-reserve stem cells (satellite cells), which then proliferate and differentiate to form new myofibers that orchestrate the muscle reparation [147,148,149]. During the remodeling phase, angiogenesis of skeletal muscle capillaries and the maturation of regenerated myofibres occur, restoring muscle metabolism and contraction functions [149,150,151,152].

### 8.1. Roles of PPARβ/δ Regulation in Satellite Cells during Muscle Regeneration

After an injury, satellite cells, as the main adult muscle stem cells, get activated and provide an indispensable role during muscle regeneration [153,154]. The satellite cells and their progeny expand as myogenic precursor cells, where most commit towards terminal differentiation and fuse with existing myofibres, so as to regenerate and restore functional myofibers [154]. A small percentage of these myogenic precursor cells, which do not commit into terminal differentiation, return to a quiescent state, providing a pool of satellite cells so as to sustain the muscle’s capacity for future regeneration [155]. Satellite cells are notoriously difficult to study, because of their low abundance under the basal lamina of skeletal muscle. Currently, knowledge of human satellite cells is limited, and most of the studies of satellite cells are performed using mice models [155].

PPARβ/δ has been shown to be important for the proper maintenance of satellite cells, as well as postnatal muscle myogenesis, and it is better studied among the PPAR proteins, because of its abundant expression in skeletal muscle. The specific ablation of PPARβ/δ in the mouse satellite cells has been reported, with approximately 40% fewer satellite cells than their wild-type littermates [156]. A similar observation was also reported in total PPARβ/δ-knock out mice [157]. Mice with PPARβ/δ-deficient muscle progenitor cells exhibited impaired muscle regeneration after cardiotoxin-induced injury and exhibited reduced growth kinetics and proliferation in primary cultures [156]. Furthermore, these mice developed metabolic syndrome upon aging, similar to the PPARβ/δ knockout mice [43,156,157]. The authors found reduced foxhead box protein (FOXO1) expression in quiescent PPARβ/δ-deficient satellite cells, which impaired the proliferation and differentiation ability of these satellite cells during muscle regeneration, thus suggesting that PPARβ/δ regulates the regenerative capability of skeletal muscle through FOXO1 [156]. In addition, CPT1β expression was also found to be reduced during quiescence , but the differences were abolished on day 5 of muscle regeneration [156], suggesting a possible PPARβ/δ-regulated metabolic role during quiescence [156].

Recent findings on the role of the lipid and glucose metabolism in stem cell cellular homeostasis have been increasingly postulated to be vital in stem cell maintenance and their proliferative activity [158,159]. Delineation of cellular metabolism in satellite cell fate could potentially offer pharmacological strategies in the treatment of degenerative muscle diseases, such as Duchenne muscular dystrophy (DMD). PPARβ/δ has been suggested as a direct transcriptional regulator of utrophin A, a key member of the dystrophin-associated protein complex [160,161]. The expression of utrophin A, stimulated by the PPARβ/δ agonist, GW501516, in the mdx mouse model of DMD has been shown to improve sarcolemma integrity, protect muscles from contraction-induced damage, and help to alleviate muscle wasting, which ultimately slowed down the disease progression [161]. Therefore, understanding the function of PPARβ/δ, and potentially the two other PPAR members, in skeletal muscle progenitor cells has important implications for muscle regeneration and the treatment of degenerative muscle diseases.

### 8.2. PPAR-Regulated Paracrine Networks between Muscle and Other Cell Types

Inflammation, specifically the infiltration of macrophages during early phases of muscle regeneration, is a major component for efficient healing and repair. Varga et al. [162] showed that myeloid-specific conditional PPARγ knockout mice exhibited a pronounced delay in muscle regeneration following a toxin-induced injury, compared with their wild-type counterparts. The injured muscle in these mice displayed a reduced muscle differentiation without differences in macrophage infiltration and phagocytic activity. They determined that the macrophage secretion of growth differentiation factor 3 (GDF3), through a direct PPARγ regulation, is a potent inducer of myotube formation, demonstrating the role of PPARγ-dependent paracrine signaling between the infiltrated macrophages and regenerating muscle [162].

Skeletal muscle is known to be highly vascularised, and numerous studies have demonstrated the importance of myogenesis and angiogenesis during skeletal muscle regeneration [163,164,165]. Recent findings on the PPARβ/δ-modulated paracrine network between the endothelial progenitor cells and regenerating myofibers, have been reported to promote both myogenesis and capillary angiogenesis [165]. PPARβ/δ activation in endothelial progenitor cells promotes insulin-like growth factor 1 (IGF1) signaling pathway in both the skeletal muscle and endothelial cells, via a direct PPARβ/δ induced transcriptional activation of matrix metalloproteinase 9 (MMP9) [165]. Matrix metalloproteinases are well known for their proteolytic activities in the extracellular matrix and they promote angiogenesis [166]. The increased MMP9 secretion from PPARβ/δ agonist-treated endothelial progenitor cells, promotes the (MMP9)-mediated insulin-like growth factor-binding protein 3 (IGFBP3) proteolysis, and thereby modulates the IGF1 activity [165,167]. The MMP9-dependent increase in IGF1 signaling was further demonstrated via the transplantation of PPARβ/δ-activated endothelial progenitor cells to a hindlimb ischaemic mice model. These mice showed an increase in regenerating the myofiber numbers and an enhanced capillary-to-myocyte ratio. The enhanced muscle regeneration and increased angiogenesis promoted a better muscle architecture with reduced fibrosis, and thereby protected the ischaemic limb from hypoxic damage [165].

Interestingly, recent reports on adiponectin produced by skeletal muscle as a myokine, exert anti-diabetic metabolic effects similar to PPAR activation [168]. The skeletal muscle-derived adiponectin has been demonstrated to regulate the fatty acid metabolism, increase glucose uptake, and induce mitochondrial biogenesis, through human skeletal muscle primary culture, muscle biopsies, and gain/loss function studies in rodent models [169,170,171,172]. Adiponectin promotes fatty acid uptake and oxidation through a series of sequential activation, involving AMPK, p38 mitogen-activated protein kinase (MAPK), and PPARα. In skeletal muscle, the activation of AMPK has been known to inhibit lipid biosynthesis through the phosphorylation of acetyl-CoA carboxylase (ACC) [173,174]. Indeed, adiponectin treatment in mouse myotube inhibited ACC phosphorylation in a time-dependent manner [170]. The PPARγ agonist, rosiglitazone, has been shown to induce adiponectin production and secretion directly [175], and is directly correlated with the rosiglitazone-mediated improvement in insulin sensitivity [176]. The overexpression of PPARγ in the mouse skeletal muscle also increased adiponectin expression, which protected these mice from high-fat diet induced insulin resistance [117].

## 9. Regulation of PPARs during Aging

Both physical exercise and aging are two physiological situations that have marked, but opposite, effects on muscle mass. Aging is a complex and multifactorial process that is characterized by progressive, endogenous, and irreversible alterations in cellular signaling, and it is associated with the slow and concerted decline of physiological functions [177]. Moreover, age is the single most significant risk factor for metabolic disorders, such as obesity, T2DM, and other major debilitating and life-threatening conditions [178]. In humans, aging leads to a loss of muscle mass, though the magnitude of loss varies substantially among individuals [179]. Age-related muscle loss is also accompanied by fiber type transformation, metabolic changes, and ectopic fat accumulation over time [180]. In aged muscles, type II glycolytic fibers, particularly type IIx, are susceptible to both atrophy and fiber type switching [180]. Compared to the percentage of glycolytic fibers, an increased percentage of oxidative fibers has been reported in the elderly [181]. Although type I muscle fiber size is largely unaffected [182,183], lower maximal force generation by type I and type IIa fibers was observed in older men, in comparison to that of the similar fibers in younger men [184].

### Evidence for the Involvement of PPARs during Aging

In aged muscles, all three PPAR expression levels are decreased and contribute to carbohydrate-lipid metabolism dysregulation, reduced muscle regeneration, and fiber remodeling [185,186,187]. In addition, the PGC-1α expression levels, as well as both the oxidative and glycolytic enzymatic capacity, are compromised in the aged skeletal muscle. The age-related decreases in fat oxidation have been consistently associated with reductions in both the quantity and the oxidative capacity to metabolize fats [188]. Lipid metabolism may be further impaired because of the increased lipid accumulation in aged muscle [189]. The decrease in both myonuclear density and mitochondria numbers in aged muscle has been associated with PPARβ/δ deficiency [43,186]. In rodents, PPARβ/δ overexpression and pharmacological activation stimulate nuclei accretion through the fusion of pre-existing muscle precursor cells to myofibers [186,190]. PPARβ/δ agonist treatment in aged mice restores the muscle fiber distribution profile and the oxidative capacity of the fast-twitch fibers, similar to those of the young untreated counterparts [186].

PPARα may play a role in glucose utilization in aged muscle. In PPARα knockout mice, an age-dependent reduction in glycolysis has been observed in the soleus muscle, which comprises mainly of slow-twitch type I fibers [185]. In addition, decreased muscle glycogen concentrations have been detected in aged PPARα-deficient mice. This suggests a role for PPARα in modulating metabolic changes during the normal aging process. Interestingly, the clinical use of fibrates may cause muscle weakness and pain (myopathy), or rhabdomyolysis in rare cases [191]. The exact mechanism of PPARα activation in diseased and aged skeletal muscle remains unclear. However, the mechanism may be partly mediated by the increased oxidative stress and tissue damage associated with PPARα-induced activity [192,193].

Aging is associated with progressive declines in both insulin sensitivity and glucose tolerance [194,195]. These effects are partly caused by decreased insulin production by the pancreatic islets and deregulated insulin signaling in muscle [196]. The PPARγ and GLUT4 expression levels are reduced in the skeletal muscle of aged rodents and humans [187,197,198]. In middle-aged adults with both diabetic and non-diabetic histories, insulin-sensitizing TZD compounds improve insulin sensitivity and glucose tolerance, and increase the likelihood of regression from pre-diabetes to normal glucose regulation [199,200]. In aged rodents, rosiglitazone treatment reverses age-related alterations in plasma triglyceride and glucose levels [201]. Paradoxically, in aged animals, mice that were heterogenous for PPARγ displayed greater insulin sensitivity than their wild-type counterparts [202]. This increased insulin sensitivity was lost upon TZD treatment or high-fat diet administration [203]. The authors suggest that PPARγ deficiency partially protects from normal physiological age-induced decreases in insulin sensitivity. In short, the physiological impact and role of diminished PPARγ expression in insulin resistance during the aging process are not clearly understood.

Although PPAR activation has beneficial effects on various metabolic dysfunctions, its beneficial effects on the aging process are not fully understood. More importantly, given the complexity of aging, there are other factors that contribute to aging that have not been discussed here. However, increasing evidence demonstrates that countermeasures can improve age-related metabolic syndromes and muscle loss, partially through modulating endogenous PPAR expression. In addition to pharmacological PPAR activation, interventions such as exercise have been shown to preserve muscle integrity in both aging humans and rodent models. The molecular changes in both lipid and glucose metabolism, after a single bout of exercise in aged humans, have been reported to increase skeletal muscle insulin action [204]. The loss of muscle mass not only reduces mobility and functional capacities which affect the quality of life, but also increases the risks associated with falls and age-related diseases. Developing treatments for age-related and disease-related muscle loss may improve the active life expectancy of older adults, thus leading to substantial health-care savings and an improved quality of life.

## 10. Concluding Remarks and Perspectives

Numerous studies have provided compelling evidence for important roles of PPAR in skeletal muscle physiology. The capacity to modulate PPAR activity with appropriate agonists or antagonist, further underscores their potential as therapeutic targets. However, the widespread use of these ligands is plagued by their accompanying side effects. Beside myopathy, fibrates are also known to increase the risk for gallstones formation [205] and renal failure [206]. The safety reputation of TZDs suffered as well when the extended use of rosiglitazone and pioglitazone were associated with an increased risk of heart attack/stroke and bladder cancer [207,208]. Although drugs for PPARβ/δ have not been clinically approved, the selective agonist GW501516 has been sold illegally as an endurance booster by its online supplement name, endurobol. GW501516 has been included in the banned substance list since 2009 by the World Anti-Doping Agency, and was re-categorized as a ‘hormone and metabolic modulator’ drug in 2012. The clinical development of GW501516 was halted in 2007 after increased incidences of several cancer types were observed in rodents [209]. Recent developments in dual- and pan-PPAR agonists displayed therapeutic benefits for the complex and wide-range metabolic disorders [8]. One example is saroglitazar, a dual PPARα/γ agonist, currently approved in India for the treatment of T2DM and dyslipidemia. Thus, the pharmacological effort in the development of combined PPARs therapeutic effects, with reduced side effects, will be crucial for next-generation drug candidates for metabolic disorders.

Skeletal muscle has been identified as an endocrine organ that expresses and releases myokines as messengers among different organs, as well as within the muscle itself. There are limited studies on the effect of PPAR on the expression of myokines, and even fewer studies on the reciprocal effect of myokines on PPAR expression and activity. For example, the expression of angiopoietin-like 4 (ANGPTL4) is an exercise-responsive myokine and is regulated by PPARs [210,211]. ANGPTL4 may regulate the lipoprotein lipase-dependent plasma clearance of triglyceride from the skeletal muscle during exercise. Another prominent PPAR-regulated myokine is interleukin-6 (IL6), whose expression can be paradoxically exercised-induced or increased during obesity and T2DM [212]. The exact mechanistic involvement of muscle-derived IL6 in health and disease, however, remains elusive, and almost nothing for the IL6 autocrine feedback regulation on PPAR. It is conceivable that pharmacological compounds that mimic the benefits of exercises will also be helpful for elderly adults, as well as for individuals with poignant mobility impairment [213].

The impact of gut microbiota on the whole-body physiology is beginning to be recognized. The bidirectional signaling between the gut microbiota and the brain has been shown to influence neurotransmission and alter behavioral responses through the changes of microbiota-derived metabolites composition. One of the dominant gut-derived metabolites are the short chains fatty acids, such as acetate and propionate, which have been shown to strongly exhibit anti-lipolysis activity in the adipose tissue [214,215].

The gut microbiota and their metabolites or components can modulate the immune system, based on their translocation into tissues and the circulatory system [216]. In recent years, the gut microbiota has been implicated in altered skeletal muscle fiber type proportions in obese porcine, offering a new perspective on the development of dietary supplements for muscle maintenance and regeneration [217]. However, the biological impact, as well as the cause and effect of this gut-muscle connection, remains to be fully understood.

In conclusion, it is clear that PPARs play an essential role in regulating energy homeostasis in skeletal muscle. It is foreseeable that, with a new development in drug design and a better understanding of PPAR’s relationship with myokines, among others, PPARs remain important pharmaceutical targets for the therapeutic strategies in order to combat different facets of metabolic syndrome.

## Abbreviations

acetyl-CoA	acetyl-coenzyme A
AF1	activation function 1
AF2	activation function 2
AKT/PKB	protein kinase B
AMPK	AMP-activated protein kinase
ANGPTL4	angiopoietin-like 4
ATGL	adipose triglyceride lipase
BMI	body mass index
CD36	cluster of differentiation 36/SR-B2
CPT1	carnitine palmitoyltransferase I
CRY1	Cryptochrome 1
DBD	DNA binding domain
DGAT1	diacylglycerol acyltransferase 1
ER	endoplasmic reticulum
FABP3	fatty acid binding protein 3
FATP	fatty acid transport protein
FATPc	cytoplasmic FABP
FATPpm	plasma membrane FABP
GLUT4	glucose transporter 4
HSL	hormone-sensitive lipase
IGFBP3	insulin-like growth factor-binding protein 3
IL6	interleukin-6
IMCL	intramyocellular lipid
IRS1	insulin receptor substrate 1
LBD	ligand binding domain
LD	lipid droplets
LPL	lipoprotein lipase
MAG	monoacylglycerol
MAPK	mitogen-activated protein kinase
MGL	monoacylglycerol lipase
NRF	nuclear respiratory factor
PDC	pyruvate dehydrogenase complex
PDK	pyruvate dehydrogenase kinase
PGC-1	PPARγ-coactivator 1
PI3K	phosphatidylinositol-3 kinase
PKC	protein kinase C
PLIN	perilipin
PP2A	protein phosphatase 2A
PPAR	peroxisome proliferator-activated receptor
PPRE	peroxisome proliferator response element
RXR	retinoid X receptors
SCD	stearoyl-CoA desaturase
T2DM	type 2 diabetes mellitus
TA	tibialis anterior
TCA	tricarboxylic acid
TFAM	mitochondrial transcription factor A
TFB1M	mitochondrial transcription factors B1
TFB2M	mitochondrial transcription factors B2
TZD	Thiazolidinediones
